# The role of kappa opioid receptors in stress-induced reinstatement of alcohol seeking in rats

**DOI:** 10.1002/brb3.222

**Published:** 2014-02-17

**Authors:** Douglas Funk, Kathleen Coen, A D Lê

**Affiliations:** 1 Neurobiology of Alcohol Laboratory, Centre for Addiction and Mental HealthToronto, Canada; 2 Department of Pharmacology, University of TorontoToronto, Canada; 3 Department of Psychiatry, University of TorontoToronto, Canada

**Keywords:** Alcohol self-administration, conditioned cue, CRF receptors, kappa opioid receptors, reinstatement, stress

## Abstract

**Introduction:**

Stress is related to heavy alcohol use and relapse in alcoholics. Using the reinstatement model, we have shown that corticotropin-releasing factor (CRF) underlies stress-induced relapse to alcohol seeking in laboratory rodents. Little is known about how other neurotransmitters interact with CRF in these effects. Dynorphin and its receptor (kappa opioid receptor, KOR) are involved in stress responses and in alcohol seeking. KOR and CRF receptors (CRF R) may interact in the production of stress-related behaviors but it is not known whether this interaction is involved in reinstatement of alcohol seeking.

**Methods:**

Male Long Evans rats were trained to self-administer alcohol (12% w/v). After extinction of responding, we determined the effects of the KOR agonist, U50,488 (2.5, 5 mg/kg) on reinstatement of alcohol seeking, and their sensitivity to the selective KOR antagonist nor-binaltorphimine dihydrochloride (nor-BNI) (10 mg/kg) administered at different times before U50,488. We then examined the effects of nor-BNI on reinstatement induced by the stressor yohimbine (1.25 mg/kg) and on reinstatement induced by exposure to alcohol-associated cues. Finally, we determined whether CRF R1 blockade with antalarmin (10, 20 mg/kg) attenuates alcohol seeking induced by U50,488.

**Results:**

U50,488 reinstated alcohol seeking. Prior treatment with nor-BNI 2, but not 24 h before administration of U50,488 or yohimbine blocked reinstatement induced by these drugs. Cue-induced reinstatement was blocked by nor-BNI administered 2 h prior to testing. Finally, U50,488-induced reinstatement was blocked by antalarmin.

**Conclusions:**

These data further support a role for KOR in reinstatement of alcohol seeking under nonstress and stressful conditions and that KOR and CRF R interact in these effects.

## Introduction

The major problem in the treatment of alcoholism is relapse (Dawson et al. 2007). Studies using correlational methods in humans suggest that stressful life events are positively related to heavy alcohol use and relapse (Brown et al. 1995; Sinha and Li 2007). We and others have used the reinstatement model to study the mechanisms underlying the effects of stress on relapse in laboratory rodents (Le and Shaham 2002b; Mason et al. 2009). With it, we were the first to show that intermittent footshock and the *α*-2 adrenoceptor antagonist yohimbine which induces craving in alcoholics, reinstates extinguished responding for alcohol in rats (Le et al. 2005, 2009, 2011; Marinelli et al. 2007a). Other studies in our laboratory and elsewhere have established that the stress-related peptide corticotropin-releasing factor (CRF) is a critical mediator of relapse to alcohol induced by stressors, including yohimbine and footshock (Le et al. 2000; Marinelli et al. 2007a). Recent work has begun to determine how CRF may interact with other neurotransmitters in stress-related behaviors such as anxiety and place aversion. Converging lines of evidence show that one such neurotransmitter, the endogenous opioid dynorphin (DYN) and its receptor (kappa opioid receptor, KOR) are involved in responses to stress (McLaughlin et al. 2006; Land et al. 2008) and in motivation to seek alcohol and other drugs (Holter et al. 2000; Valdez et al. 2007; Walker et al. 2011; Schank et al. 2012). Although KOR and CRF receptors (CRF R) have been shown to interact in stress-related behaviors (Land et al. 2008), little is known about how they may interact in stress-induced reinstatement of drug and alcohol seeking.

The endogenous opioids comprise *β*-endorphin, the enkephalins and DYN that, respectively, act at mu, delta, and KOR. These receptors show distinctive patterns of localization in the central nervous system, including areas implicated in drug seeking and responses to stress (Mansour et al. 1994). The involvement of mu and delta receptors in drug and alcohol seeking is well-established (Gianoulakis et al. 1996; Pradhan et al. 2011). We and others have studied their role in reinstatement of alcohol seeking induced by exposure to discrete and contextual cues (Marinelli et al. 2007b, 2009, 2010). DYN and KOR have not only received less attention but have also been implicated in drug and alcohol seeking. Although the data from studies on basal alcohol consumption in DYN and KOR knock-out mice are mixed (Walker et al. 2012), pharmacological studies suggest that DYN and KOR can modulate alcohol consumption (Walker et al. 2011; Schank et al. 2012). KOR blockade also reduces spontaneous recovery of lever pressing for alcohol as well as cue-induced reinstatement of alcohol seeking (Deehan et al. 2012; Schank et al. 2012).

Stress stimulates DYN release of in brain areas involved in motivation and reward (McLaughlin et al. 2003; Shirayama et al. 2004). A number of data suggest that the released DYN is involved in stress-related behaviors. KOR antagonists attenuate the depressive effect of repeated forced swimming (Pliakas et al. 2001; Mague et al. 2003) as well as the analgesia and immobility induced by social defeat stress (McLaughlin et al. 2003, 2006; Shirayama et al. 2004). Consistent with this, DYN and KOR are involved in the effects of stress on drug and alcohol seeking. DYN knockout mice do not show stress-induced increases in alcohol drinking (Sperling et al. 2010). Antagonism of KOR blocks increased alcohol intake induced by presentation of a cue previously associated with alcohol withdrawal (Berger et al. 2013). KOR antagonists block stress-induced potentiation of the development of place preference conditioning to cocaine and alcohol, (McLaughlin et al. 2006; Sperling et al. 2010), reinstatement of place preference to cocaine (Redila and Chavkin 2008; Beardsley et al. 2010), nicotine (Jackson et al. 2012) and stress-induced reinstatement of lever pressing for cocaine (Beardsley et al. 2005) and heroin (Zhou et al. 2013). These data provide evidence that the aversive and stress-like effects produced by stimulation of KOR contribute to reinstatement of drug seeking. On the other hand, it was recently reported that KOR blockade did not affect stress-induced reinstatement of lever pressing for alcohol (Schank et al. 2012). The reasons for this discrepant finding are not known.

The interaction of KOR and CRF R has been studied in detail in the production of anxiety and aversive responses. Antagonism of KOR reduces the CRF R1-mediated anxiety induced by stress in mice (Bruchas et al. 2009). KOR antagonists block place aversion induced by CRF or a CRF R2 agonist, but KOR agonist-induced place aversion was unaffected by CRF R2 blockade (Land et al. 2008). The results of these studies clearly indicate an interaction between KOR and CRF R in stress-related behaviors.

These data lead to the hypothesis that a DYN/KOR-CRF R interaction may be involved in reinstatement of drug seeking. DYN is co-localized with CRF in brain regions implicated in drug seeking (Meister et al. 1990; Reyes et al. 2008). KOR or CRF R stimulation modifies central levels of CRF or DYN, respectively (Nikolarakis et al. 1986; Yajima et al. 1986). Local CRF infusion into the CeA increases DYN release (Lam and Gianoulakis 2011). Intracerebroventricular (i.c.v.) infusion of CRF increases KOR phosphorylation in the amygdala, which is blocked by the CRF R1 antagonist antalarmin (Bruchas et al. 2009), consistent with a CRF-induced activation of DYN neurons and DYN release. To our knowledge, however, there is only one published report specifically addressing a potential CRF-DYN interaction in drug seeking. The CRF R1 antagonist CP 154,526 blocked KOR agonist-induced reinstatement of cocaine seeking in squirrel monkeys (Valdez et al. 2007). The potential that CRF interacts with DYN in reinstatement of alcohol seeking has not been addressed.

In the present studies, we, therefore, explore the role of KOR, and their interaction with CRF in the reinstatement of alcohol seeking. We will first conduct a dose-response analysis of the effects of the selective KOR agonist, U50,488 on reinstatement of alcohol seeking and whether its effects are blocked by the selective KOR antagonist, nor-binaltorphimine dihydrochloride (nor-BNI). We will then examine the effects of nor-BNI on reinstatement induced by the pharmacological stressor yohimbine, a drug that we have shown to induce reinstatement in a CRF R1-dependent manner. Nor-BNI will be administered at two different pretreatment times prior to U50,488 or yohimbine, as its selectivity for KOR versus other opioid receptors has been shown to vary in a time-dependent manner. Then we will examine whether reinstatement of alcohol induced by cues previously associated with alcohol seeking is blocked by nor-BNI. Finally, we will determine whether blockade of CRF R1 with antalarmin attenuates alcohol seeking induced by U50,488.

## Material and Methods

The experimental procedures followed the “Guide for the care and use of laboratory animals” (Canadian Council on Animal Care, 1993) and were approved by the animal care and use committee of the Centre for Addiction and Mental Health.

### Animals

Long Evans rats (Charles River, St-Constant, QC, Canada) weighing 200–250 g at the start of the experiment were used. The rats were individually housed under a 12:12 h light-dark cycle (light on at 7:00 am to 7:00 pm). Food and water were freely available in the home cage at all times and the temperature was maintained at 21 ± 1°C.

### Apparatus

The alcohol self-administration chambers were constructed locally and were equipped with two levers, symmetrically centered on a side panel. During the self-administration sessions, responding on one lever (an active lever) activated an infusion pump (Razel Sci., Stamford, CT), while responding on the other lever (an inactive lever) was recorded, but did not activate the pump. Activation of the infusion pump resulted in the delivery of 0.19 mL of alcohol into a drinking receptacle located between the two levers and initiated a 5-sec timeout period. During the timeout, the house light was turned off, a key light over the active lever was turned on, and white noise was emitted by a speaker. The beginning of the sessions was signaled by the illumination of a houselight located at the top of the self-administration chamber; at the end of the session, the houselight was turned off. When alcohol was left in the drinking receptacle after a self-administration session, it was measured and the volume was taken into account for intake calculation.

### Procedure

#### Alcohol self-administration training

Rats were trained to self-administer alcohol as described previously (Le et al. 1998; Le and Shaham 2002a). Briefly, they were initially provided with access to alcohol solutions and tap water for 30 min/day in drinking cages (30 × 18 × 18 cm) containing Richter tubes. Alcohol solutions were provided in increasing concentrations: 3% (w/v) for the first 5 days, 6% (w/v) for the next 5 days and 12% (w/v) for the next 10 days. Subsequently, operant self-administration of alcohol (12%) was initiated in 1-h daily sessions on a fixed ratio-1 (FR-1) 5-sec timeout reinforcement schedule for at least 5 days (1 h/day). The requirement for alcohol delivery was then increased to FR-2 for 5 days and then to FR-3 for at least 6 days, until the rats demonstrated 3 days of stable alcohol-taking behavior (variability of less than 20% of the mean). Animals that did not consume 0.4 g/kg alcohol during the limited access training conditions were excluded from analysis. We have found that animals that consume less than 0.4 g/kg are difficult to train to self-administer alcohol. Furthermore, stable and high lever responding is critical in order to achieve a robust and reliable reinstatement effect. In the present experiments, about 85% of the animals achieved these criteria and were successfully trained to self-administer alcohol.

#### Extinction of alcohol-reinforced behavior

The experimental procedures during the extinction sessions were the same as those during the self-administration sessions, with the exception that responding on the active lever did not lead to alcohol delivery, and the cue lights and speakers signaling delivery were disconnected. Tests for reinstatement commenced after 7–12 extinction sessions, after the rats reached the extinction criterion of fewer than 12 presses on the previously active lever during the 1 h session. During the last four extinction sessions prior to testing the rats received i.p. water vehicle injections to habituate them to the injection procedures.

#### Test for reinstatement of alcohol seeking

In Experiments 1, 2, 3, and 5, tests were conducted under the same conditions experienced during extinction. In Experiment 4, active lever presses made by the animals resulted in presentation of the cues that were previously associated with alcohol delivery during self-administration training.

### Drugs

Alcohol solution for oral self-administration was prepared by diluting 95% ethanol in tap water. U50,488, nor-BNI, and yohimbine HCl were dissolved in distilled water, while antalarmin was suspended in 10% cremophor in saline; drugs were injected i.p. in a volume of 1 mL/kg. Nor-BNI, U50,488, and antalarmin were obtained from the NIDA Drug Supply Program (Baltimore, MD) and yohimbine was obtained from Sigma-Aldrich (St. Louis, MO). The doses of yohimbine and antalarmin and their pretreatment times were based on our published studies (Le et al. 2005, 2009; Marinelli et al. 2007a) and those for U50,488 and nor-BNI were based on previous reports (Redila and Chavkin 2008; Schank et al. 2012).

### Statistical analysis

Statistical analyses were performed separately on the numbers of responses made on the previously active and inactive levers during the reinstatement tests. Data were analyzed with analysis of variances (ANOVAs) and significant interactions (*P* < 0.05) were followed by Newman-Keuls post hoc tests.

### Experiment 1: Effects of U50,488 on reinstatement

Twelve rats were trained to self-administer alcohol and received extinction sessions as described above. A within design was used with U50,488 dose (vehicle, 2.5 and 5.0 mg/kg, i.p.) as the factor. Once the extinction criterion was reached, rats received vehicle (water) or one of the doses of U50,488, 30 min before 1-h reinstatement test sessions conducted in the operant chambers, in counterbalanced order with at least 2 days between each test. On the days between the drug tests, rats were injected i.p. with water and received drug-free extinction sessions.

### Experiment 2: Effect of nor-BNI on reinstatement of alcohol seeking induced by U50,488

Twenty-three rats (*n* = 7–8 per group) were trained to self-administer alcohol and received extinction sessions. In this and in the following experiments, rats were assigned to matched groups based on alcohol intake and extinction responding. A mixed design was used with the between factor of nor-BNI pretreatment condition (vehicle, nor-BNI 2 h, nor-BNI 24 h) and within factor of U50,488 pretreatment condition (vehicle, 5 mg/kg, i.p.). One group of animals received injections of nor-BNI vehicle or nor-BNI (10 mg/kg, i.p.) 2 h before the U50,488 vehicle or U50,488 injections. The other group received nor-BNI 24 h prior to U50,488 vehicle or U50,488 injections. Thirty minutes after the U50,488 vehicle or U50,488 injections, rats were placed in the operant chambers for the 1-h reinstatement test session. In order to minimize the use of animals in Experiments 2 and 3, we did not include a 24 h nor-BNI vehicle condition; we have found that baseline extinction responding is extremely stable from day to day once the extinction criterion is reached.

### Experiment 3: Effect of nor-BNI on yohimbine-induced reinstatement of alcohol seeking

The effects of nor-BNI on yohimbine-induced reinstatement was assessed in 36 rats (*n* = 12 per group), trained to self-administer alcohol and their responding was extinguished as above. A mixed design was used with the between factor of nor-BNI pretreatment condition (vehicle, nor-BNI 2 h, nor-BNI 24 h) and within factor of Yohimbine condition (vehicle, 2.5 mg/kg, i.p.). During testing, each rat was injected with the nor-BNI vehicle 2 h before, or nor-BNI (10 mg/kg, i.p.), 2 or 24 h prior to injections of the yohimbine vehicle (water) or yohimbine. Forty-five minutes after the vehicle or yohimbine injections, rats were placed in the operant chambers for the 1-h reinstatement test session.

### Experiment 4: Effect of nor-BNI on reinstatement of alcohol seeking by alcohol-associated cues

Twenty-two rats (*n* = 11 per group), trained to self-administer alcohol and their responding extinguished as above, were used to determine the effects of nor-BNI on reinstatement induced by the light/tone cue previously associated with alcohol self-administration. A mixed design was used with the between factor of nor-BNI pretreatment condition (vehicle, nor-BNI 2 h) and within factor of Cue condition (No cue, Cue). During testing, each rat was injected with the nor-BNI vehicle or nor-BNI (10 mg/kg, i.p.) 2 h prior to 1-h test sessions. Rats were placed in the operant chambers and received one non-contingent presentation of the tone-light cue. Subsequent presses on the active lever resulted in cue presentation on a FR1 schedule during the remainder of the session. Extinction responding without cue presentation the previous day was used as the baseline. A 24 h nor-BNI condition was not included as such pretreatment did not affect U50,488- and yohimbine-induced reinstatement in Experiments 2 and 3.

### Experiment 5: Effect of antalarmin on U50,488-induced reinstatement of alcohol seeking

Twenty-four rats (*n* = 12 per group) were trained and their responding was extinguished as above and they were used to assess the effect of antalarmin on U50,488-induced reinstatement. A mixed design was used with the between factor of U50,488 dose (vehicle, 5 mg/kg, i.p.) and within factor of Antalarmin dose (vehicle, 10, 20 mg/kg, i.p.). On the test days, each rat was injected with the antalarmin vehicle (10% cremophor in saline) or one of the doses of antalarmin, and 30 min later with vehicle or U50,488. Thirty minutes after the vehicle or U50,488 injections, rats were placed in the operant conditioning chambers for a 1-h reinstatement test session. There were at least 2 days between drug tests, and on these days animals were injected with water, i.p. and received extinction sessions.

## Results

**Figure 1:**
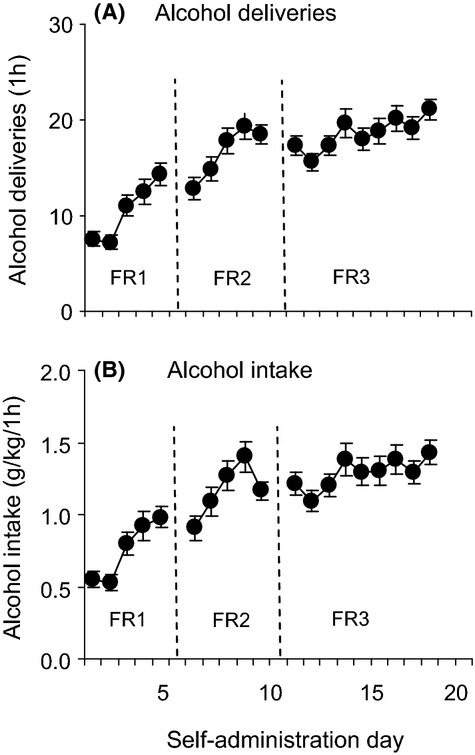
Alcohol self-administration. (A) Alcohol deliveries and (B) intake during self-administration training at fixed ratio (FR) 1, 2, and 3. The self-administration days at the different FRs are separated by dashed lines. Data are presented as means (±SEM). *n* = 117.

Figure 1 shows the mean number of alcohol deliveries (A) and intake in g/kg (B) during alcohol self-administration training of the animals in the experiments. The average numbers of active lever responses (±SEM) made over the last 3 days of alcohol self-administration were 82.78 ± 4.12.

### Experiment 1: Effects of U50,488 on reinstatement

**Figure 2:**
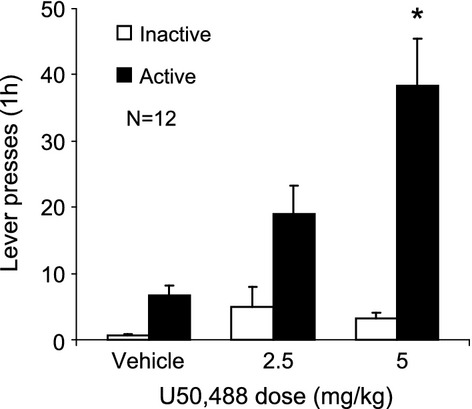
Effect of the KOR agonist U50,488 on reinstatement of alcohol seeking. Male Long Evans rats were trained to self-administer alcohol (12%, 0.19 mL/delivery, 1 h/day) to FR-3. Responding was then extinguished in daily 1 h sessions. Vehicle or U50,488 (2.5, 5 mg/kg) was administered i.p. 30 min prior to the test session in counterbalanced order with at least 2 days separating each dose. The data are means ± SEM. *Different from vehicle (*P* < 0.05).

Figure 2 shows the effects of U50,488 on reinstatement of alcohol seeking. The one-way repeated measures analysis of active lever responding showed a significant effect of U50,488 dose [*F*(2,22) = 10.33, *P* < 0.05]. The effects of U50,488 were significantly different from vehicle at the 5 mg/kg dose [*P* < 0.05], but not at the 2.5 mg/kg dose (*P* > 0.05). There were no significant effects of U50,488 on responding on inactive lever responding (Table 1).

**Table 1:** Inactive lever pressing in each of the experiments

*Experiment 1*		
U50,488 dose		
Vehicle	0.67 ± 0.28	
2.5 mg/kg	5.0 ± 3.07	
5 mg/kg	3.25 ± 0.95	
*Experiment 2*		
Nor-BNI group	Baseline	U50,488
Vehicle	1.0 ± 0.45	6.4 ± 2.91
10 mg/kg-2 h	1.88 ± 0.95	1.63 ± 1.49
10 mg/kg-24 h	2.57 ± 1.49	5.86 ± 2.54
*Experiment 3*		
Nor-BNI group	Baseline	Yohimbine
Vehicle	1.25 ± 0.60	2.33 ± 0.91
10 mg/kg-2 h	0.60 ± 0.34	2 ± 0.89
10 mg/kg-24 h	0.20 ± 0.20	3.1 ± 1.37*
*Experiment 4*		
Nor-BNI group	Baseline	Cue
Vehicle	2.27 ± 0.45	2.73 ± 0.86
10 mg/kg-2 h	1.91 ± 0.90	4.82 ± 2.72
*Experiment 5*		
Antalarmin dose	Vehicle	U50,488
Vehicle	1.42 ± 0.71	2.78 ± 0.85
10 mg/kg	1.17 ± 0.64	2.67 ± 0.65
20 mg/kg	2.67 ± 1.42	4.44 ± 1.30

* Significant effect of yohimbine condition (*P* < 0.05).

### Experiment 2: Effects of nor-BNI on U50,488-induced reinstatement of alcohol seeking

**Figure 3:**
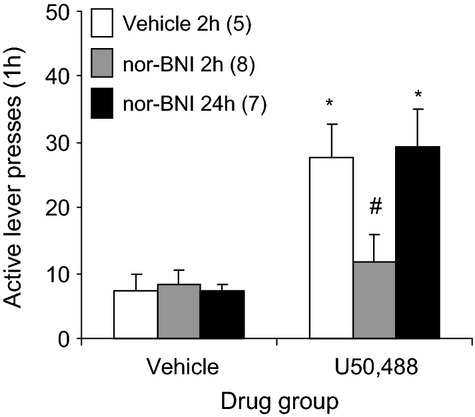
Effect of the KOR antagonist nor-BNI on U50,488-induced reinstatement of alcohol-seeking. Male Long Evans rats were trained to self-administer alcohol and their responding was extinguished. Vehicle was administered i.p. 2 h before, and nor-BNI (10 mg/kg) was administered 2 or 24 h prior to U50,488 (5 mg/kg) or its vehicle. Thirty minutes later, animals received a 1-h reinstatement test. The data are means ± SEM. Numbers in parentheses are the group NS. *Different from vehicle condition, (*P* < 0.05). ^#^Different from vehicle 2-h condition (*P* < 0.05).

Figure 3 shows the effects of nor-BNI on U50, 488-induced alcohol seeking (Fig. 3). Mixed ANOVA of active lever responding with the between factor of nor-BNI pretreatment condition and within factor of U50,488 pretreatment condition revealed a significant interaction [*F*(2,17) = 3.9, *P* < 0.05]. In animals treated with nor-BNI vehicle, U50,488 significantly increased active lever pressing compared to those administered its vehicle (*P* < 0.05). The U50,488-induced increase was blocked by nor-BNI when it was injected 2 h (*P* < 0.05), but not 24 h prior to U50,488 (*P* > 0.05). Inactive lever pressing was not significantly affected by U50,488 or nor-BNI (Table 1).

### Experiment 3: Effect of nor-BNI on yohimbine-induced reinstatement of alcohol seeking

**Figure 4:**
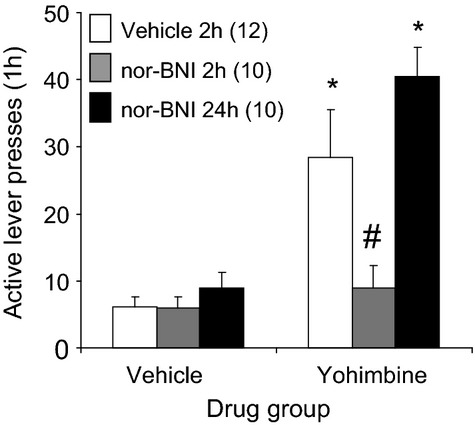
Effect of the KOR antagonist nor-BNI on yohimbine-induced reinstatement of alcohol seeking. Male Long Evans rats were trained to self-administer alcohol and their responding was extinguished. Vehicle was administered i.p. 2 h before, and nor-BNI (10 mg/kg) was administered 2 or 24 h prior to yohimbine (1.25 mg/kg) or its vehicle. Animals received a 1-h reinstatement test 30 min after the vehicle or yohimbine injection. The data are means ± SEM. Numbers in parentheses are the group NS. *Different from vehicle (*P* < 0.05). ^#^Different from vehicle 2-h condition (*P* < 0.05).

Figure 4 shows the effects of nor-BNI on reinstatement of alcohol seeking induced by yohimbine. The mixed ANOVA on active lever pressing with the between factor of nor-BNI pretreatment condition and within factor of Yohimbine condition showed a significant interaction [*F*(2,28) = 5.6, *P* < 0.05]. In animals administered the vehicle for nor-BNI, yohimbine significantly reinstated alcohol seeking (*P* < 0.05). This reinstatement was significantly reduced when nor-BNI was administered 2 h prior to yohimbine (*P* < 0.05), but not when nor-BNI was administered 24 h before. Analysis of inactive lever data showed a significant effect of Yohimbine condition, due to the fact that inactive responding was overall slightly higher in animals administered yohimbine. This effect achieved statistical significance in the 24 h nor-BNI group (*P* < 0.05). (Table 1).

### Experiment 4: Effect of nor-BNI on cue-induced reinstatement of alcohol seeking

**Figure 5:**
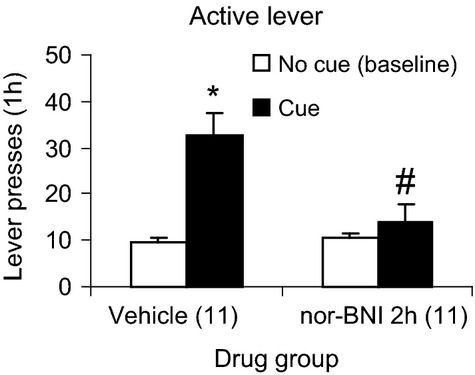
Effect of the KOR antagonist nor-BNI on cue-induced reinstatement of alcohol seeking. Rats were trained to self-administer alcohol and their responding was extinguished. Nor-BNI (10 mg/kg) was administered i.p. 2 h prior to a 1 h test of cue-induced reinstatement. Numbers in paretheses are the group NS. The data are means ± SEM. *Different from no cue condition (*P* < 0.05). ^#^Different from the cue-vehicle condition (*P* < 0.05).

Figure 5 shows the effects of nor-BNI on reinstatement induced by exposure to cues previously associated with alcohol delivery. The mixed ANOVA on active lever pressing with the between factor of nor-BNI condition and within factor of Cue condition showed a significant interaction [*F*(1,20) = 9.03, *P* < 0.05]. When animals were exposed to cues previously associated with alcohol delivery they showed a significant reinstatement of active lever pressing, compared to the no cue baseline condition (*P* < 0.05). This reinstatement was blocked in animals given nor-BNI 2 h prior to the test, compared to those administered vehicle (*P* < 0.05). There were no significant effects on inactive lever responding (Table 1).

### Experiment 5: Effect of antalarmin on U50,488-induced reinstatement of alcohol seeking

**Figure 6:**
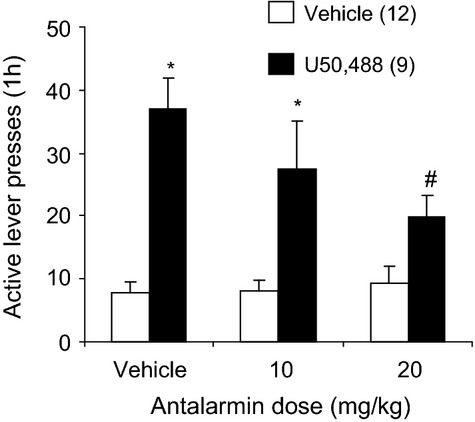
Effect of the CRF R1 antagonist antalarmin on U50,488-induced reinstatement of alcohol seeking. Male Long Evans rats were trained to self-administer alcohol and their responding was extinguished. Vehicle or antalarmin injections were given i.p. 30 min prior to vehicle or U50,488 (5 mg/kg) injections. Thirty minutes later, animals receive a 1-h reinstatement test. The data are means ± SEM, Numbers in parentheses are the group NS. *Different from U50,488 vehicle condition (*P* < 0.05). ^#^Different from antalarmin vehicle-U50,488 condition (*P* < 0.05).

Figure 6 shows the effects of antalarmin on reinstatement of alcohol seeking induced by U50,488. Mixed ANOVA with the between factor of U50,488 dose and within factor of Antalarmin dose on active lever presses revealed a significant interaction [*F*(2,38) = 3.46, *P* < 0.05]. Compared to animals treated with the vehicle, those administered U50,488 showed a significant reinstatement of alcohol seeking (Fig. 6). This reinstatement was significantly reduced by the administration of antalarmin at a dose of 20 mg/kg (*P* < 0.05), but not by 10 mg/kg. There were no significant effects of either of the drugs on inactive lever responding (Table 1).

## Discussion

These studies were done to determine the role of KOR in reinstatement of alcohol seeking under nonstress and stressed conditions, and if KOR interacts with CRF R1 in these effects. A KOR agonist, U50,488 induced reinstatement, and, as expected, this was blocked by the selective KOR antagonist nor-BNI, when it was injected 2 h, but not 24 h before U50,488. The reinstatement induced by the pharmacological stressor, yohimbine or alcohol-associated cues were also blocked by the KOR antagonist when it was administered 2 h prior to testing. Finally, we found that U50,488-induced reinstatement of alcohol seeking was blocked by antagonizing CRF R1. These data further establish a role for KOR in stress- and cue-induced reinstatement of alcohol seeking, and indicate that KOR may participate in this by interacting with the CRF systems.

### Role of KOR in alcohol seeking

Our data offer support for previous work on the role of KOR in alcohol intake and seeking (Holter et al. 2000; Walker et al. 2011; Schank et al. 2012). We found that stimulation of KOR with U50,488 robustly and dose-dependently reinstated alcohol seeking. These effects were blocked by the selective KOR antagonist nor-BNI when it was administered 2, but not 24 h before U50,488. These results demonstrate a clear facilitatory role for central KOR-containing pathways in reinstatement of alcohol seeking and they agree with previous work showing that KOR agonists reinstate conditioned place preference to cocaine in mice (Redila and Chavkin 2008) and lever pressing for cocaine in monkeys (Valdez et al. 2007) and rats (Beardsley et al. 2010). They also agree with the positive role KOR have in alcohol intake in studies using both operant and bottle drinking methods in rodents (Holter et al. 2000; Walker et al. 2011; Schank et al. 2012).

It should be noted that Walker et al. (2011) showed a significant effect of nor-BNI only in alcohol-dependent rats, and that this occurred only after the cumulative dose of this long-acting drug reached 15 mg/kg. These results suggest that in the case of alcohol self-administration, only dependent animals a sensitive to KOR blockade, and even so, require a much higher dose of nor-BNI to reduce responding. This at least in part consistent with the observation of Schank et al. (2012) that extremely high single doses of nor-BNI (30 mg/kg) are required to reduce alcohol consumption in non-dependent animals. Taken together with our present findings, these data suggest that reinstatement induced by KOR stimulation and stress is much more sensitive to KOR blockade than ongoing self-administration in alcohol-dependent rats.

We found that the selective KOR antagonist, nor-BNI significantly reduced U50,488-induced reinstatement of alcohol seeking. This effect was noted when animals were pretreated with nor-BNI 2 h, but not 24 h prior to the U50,488 injections. This time course is unexpected, as behavioral (pain) and receptor binding studies suggest that this antagonist causes a long-lasting antagonism of KOR receptors, that lasts days or weeks, and that its selectivity for KOR plateaus at 2 h and becomes maximal at 24 h after administration (Endoh et al. 1992; Jones and Holtzman 1992; Broadbear et al. 1994). Our findings concerning the time course of the ability of nor-BNI to block U50,488- or yohimbine-induced reinstatement of alcohol seeking is consistent with recent findings by Schank et al. (2012), showing that suppression of alcohol self-administration by the KOR antagonists JDTic (10 mg/kg) or nor-BNI (30 mg/kg) occurs at 2, but not 24 h after administration. On the other hand, a recent study reported that nor-BNI attenuated yohimbine-induced reinstatement of responding 24 h after administration (Zhou et al. 2013). Furthermore, JDTic-induced suppression of spontaneous recovery of alcohol responding can occur up to 14 days after JDTic treatment (Deehan et al. 2012).

The reasons for these discrepancies in the time course of the antagonism of KOR and the suppressive actions of nor-BNI and JDTic on drug- or alcohol-related behaviors are not clear. One potential explanation is that at the 2 h post nor-BNI injection time point we use, the early-appearing MOR antagonist properties of nor-BNI may be still present (Endoh et al. 1992). This is unlikely to be the case, as the MOR blockade induced by nor-BNI occurs in the first hour after administration. By 2 h post injection, selectivity for KOR is 100-fold higher than for MOR (Endoh et al. 1992). Furthermore, we saw a complete blockade by nor-BNI of the reinstatement induced by the highly selective KOR agonist U50,488 at 2, but not 24 h.

Another possibility is that time-dependent effects are highly specific to the outcomes measured. For example, most of the data on the long-lasting effects of nor-BNI come from studies measuring pain, which is mediated by quite different systems than are motivated behaviors. Arguing against this idea, however, are recent findings that nor-BNI blocked yohimbine-induced reinstatement of heroin seeking 24 h after administration (Zhou et al. 2013), and our observation that nor-BNI was still able to significantly attenuate yohimbine-induced reinstatement of nicotine seeking 24 h after administration (Grella, Funk, Coen, Li, Lê, in revision).

### KOR and stress-induced alcohol seeking

The selective KOR antagonist, nor-BNI significantly attenuated reinstatement of alcohol seeking induced by the pharmacological stressor yohimbine. These results a support those from studies on the role of KOR and stress in the expression of CPP to other drugs. Nor- BNI blocks stress- and U50,488-induced potentiation of CPP to cocaine (Schindler et al. 2010) or alcohol (Sperling et al. 2010) as well as stress-induced reinstatement of lever pressing for cocaine (Beardsley et al. 2005), heroin (Zhou et al. 2013) or nicotine (Grella, Funk, Coen, Li, Lê, in revision) in rats. Taken together, these and our present data support the idea that KOR plays an important role in stress-induced drug seeking.

However, one study reported that KOR blockade with JDTic did not affect footshock-induced reinstatement (Schank et al. 2012). The reasons for the discrepant findings are not clear. It is unlikely that nonspecific effects of nor-BNI produced the reduction in yohimbine-induced reinstatement we observed, as Schank et al. (2012) found that a dose of nor-BNI three times higher than what we used did not affect operant responding for sucrose. It is likely that differences in the neural circuitry underlying the effects of yohimbine and footshock on reinstatement or possibly between the pharmacological characteristics of nor-BNI and JDTic underlie the lack of effect of KOR blockade on footshock stress-induced reinstatement observed by Schank et al. (2012).

A transmitter that KOR may interact with in producing these effects is noradrenaline. Noradrenaline plays a key role in stress responses (Dunn and Swiergiel 2008). The distribution of KOR and noradrenaline receptors overlap in a number of brain regions involved in reinstatement of drug and alcohol seeking (Mansour et al. 1987; Zilles et al. 1993), and KOR stimulation can affect the release of noradrenaline from terminals in forebrain areas (Grilli et al. 2009). U50,488-induced reinstatement of CPP to cocaine in mice was reduced by injections of nor-BNI into the locus coeruleus, the location of noradrenergic cell bodies that project to the hippocampus and cortex (Al-Hasani et al. 2013). KOR in the locus coeruleus has also been implicated in liability to opioid abuse (Van Bockstaele et al. 2010). Future experiments should be directed at exploring the potential interaction of noradrenaline and KOR in alcohol seeking.

Our findings of blockade of cue-induced reinstatement of alcohol seeking by nor-BNI are in agreement with those of Schank et al. (2012) who showed a significant blockade of cue-induced reinstatement of alcohol seeking by another KOR antagonist JDTic. Taken together, these data suggest that KOR play a key role in reinstatement induced by alcohol-associated cues.

Data from this study and earlier work shows that KOR are involved in stress as well as cue-induced alcohol seeking. This is consistent with the speculation that drug-associated cues are a form of stress (Karoly and Hutchison 2012). Drug-associated cues and stress have additive effects on reinstatement of alcohol and cocaine seeking (Liu and Weiss 2002; Feltenstein and See 2006). The neuronal substrates underlying cue-and stress-induced relapse overlap at a number of levels (Sinha and Li 2007), although there are also important differences (Liu and Weiss 2002).

The brain areas in which DYN release underlies cue-induced drug seeking are not known. A likely candidate is the amygdala (Johansen et al. 2011; Young and Williams 2013). This area is especially enriched in KOR (Mansour et al. 1987), and has been shown to mediate different aspects of learning and memory (Maren 1996; Dityatev and Bolshakov 2005). Future studies could be aimed at determining the effects of local application of KOR antagonists on cue-induced reinstatement of alcohol seeking.

### CRF R1 and KOR interaction in alcohol seeking

Evidence for a KOR-CRF R interaction in modulating reinstatement of alcohol seeking was also found. The CRF R1 antagonist antalarmin significantly reduced reinstatement of alcohol seeking induced by U50,488. This supports the results of Valdez et al. (2007), who found that the CRF R1 antagonist CP154,526 significantly reduced reinstatement of cocaine seeking induced by the KOR agonist spiradoline, and extends them to alcohol seeking.

The interaction of CRF R and KOR in other behavioral effects of stress (anxiety, aversion) has been studied in mice (Land et al. 2008; Bruchas et al. 2009). The increased anxiety elicited by stress (forced swim or i.c.v. CRF) shown in a separate study to be mediated by CRF R1, is reduced by KOR blockade (Bruchas et al. 2009). Consistent with this, CRF-induced KOR phosphorylation is blocked in several brain regions by pretreatment with a CRF R1 antagonist.

There is also a CRF-KOR interaction in the aversive responses elicited by stress, which involves, in contrast, CRF R2 receptors. Place aversion induced by either CRF or a CRF R2 agonist was blocked by a KOR antagonist, but KOR agonist-induced place aversion was unaffected by CRF R2 blockade (Land et al. 2008). These data on anxiety and place aversion were interpreted as suggesting that CRF induces DYN release, and the released DYN activates KOR and produces aversion or anxiety. This is consistent with the results of a microdialysis study showing that injection of CRF through an adjacent cannula evokes the release of DYN, but not vice versa in the central amygdala (Lam and Gianoulakis 2011).

Our findings of nor-BNI blockade of yohimbine-induced reinstatement are consistent with this proposed mechanism, as yohimbine produces reinstatement through a CRF R1-dependent mechanism. Our results on antalarmin-induced blockade of U50,488-induced reinstatement, however, suggest a different mechanism, with an opposite relationship between the two peptides. These latter data suggest instead that KOR stimulation evokes the release of CRF, which in turn stimulates CRF R to induce reinstatement. Antalarmin blocks the effects of this released CRF on the CRF R, thereby inhibiting the reinstatement response. A brain region in which this interaction might occur is the amygdala, a critical part of the circuitry involved in responses to stress (Johansen et al. 2011). CRF and DYN is released in these regions by stress (Funk et al. 2003; Smith et al. 2012), and it possesses binding sites for CRF R and KOR (Mansour et al. 1987; Weathington and Cooke 2012).

These data provide further support for the important role of KOR in reinstatement of alcohol seeking under nonstress and stressful conditions. They also indicate an interaction between KOR and CRF in reinstatement of alcohol seeking. Further studies are necessary to elaborate the role of KOR and CRF R in stress-induced alcohol seeking. A key experiment we intend to conduct is to examine the effect of nor-BNI on reinstatement induced by the stressor, i.c.v. CRF.

## References

[b1] Al-Hasani R, McCall JG, Foshage AM, Bruchas MR (2013). Locus coeruleus kappa-opioid receptors modulate reinstatement of cocaine place preference through a noradrenergic mechanism. Neuropsychopharmacology.

[b2] Beardsley PM, Howard JL, Shelton KL, Carroll FI (2005). Differential effects of the novel kappa opioid receptor antagonist, JDTic, on reinstatement of cocaine-seeking induced by footshock stressors vs cocaine primes and its antidepressant-like effects in rats. Psychopharmacology.

[b3] Beardsley PM, Pollard GT, Howard JL, Carroll FI (2010). Effectiveness of analogs of the kappa opioid receptor antagonist (3R)-7-hydroxy-N-((1S)-1-{[(3R,4R)-4-(3- hydroxyphenyl)-3,4-dimethyl-1-pipe ridinyl]methyl}-2- methylpropyl)- 1,2,3,4-tetrahydro-3-isoquinolinecarboxami de (JDTic) to reduce U50,488-induced diuresis and stress-induced cocaine reinstatement in rats. Psychopharmacology.

[b4] Berger AL, Williams AM, McGinnis MM, Walker BM (2013). Affective cue-induced escalation of alcohol self-administration and increased 22-kHz ultrasonic vocalizations during alcohol withdrawal: role of kappa-opioid receptors. Neuropsychopharmacology.

[b5] Broadbear JH, Negus SS, Butelman ER, de Costa BR, Woods JH (1994). Differential effects of systemically administered nor-binaltorphimine (nor-BNI) on kappa-opioid agonists in the mouse writhing assay. Psychopharmacology.

[b6] Brown SA, Vik PW, Patterson TL, Grant I, Schuckit MA (1995). Stress, vulnerability and adult alcohol relapse. J. Stud. Alcohol.

[b7] Bruchas MR, Land BB, Lemos JC, Chavkin C (2009). CRF1-R activation of the dynorphin/kappa opioid system in the mouse basolateral amygdala mediates anxiety-like behavior. PLoS ONE.

[b65] Canadian Council on Animal Care (1993). Guide to the care and use of laboratory animals.

[b8] Dawson DA, Goldstein RB, Grant BF (2007). Rates and correlates of relapse among individuals in remission from DSM-IV alcohol dependence: a 3-year follow-up. Alcohol. Clin. Exp. Res.

[b9] Deehan GA, McKinzie DL, Carroll FI, McBride WJ, Rodd ZA (2012). The long-lasting effects of JDTic, a kappa opioid receptor antagonist, on the expression of ethanol-seeking behavior and the relapse drinking of female alcohol-preferring (P) rats. Pharmacol. Biochem. Behav.

[b10] Dityatev AE, Bolshakov VY (2005). Amygdala, long-term potentiation, and fear conditioning. Neuroscientist.

[b11] Dunn AJ, Swiergiel AH (2008). The role of corticotropin-releasing factor and noradrenaline in stress-related responses, and the inter-relationships between the two systems. Eur. J. Pharmacol.

[b12] Endoh T, Matsuura H, Tanaka C, Nagase H (1992). Nor-binaltorphimine: a potent and selective kappa-opioid receptor antagonist with long-lasting activity in vivo. Arch. Int. Pharmacodyn. Ther.

[b13] Feltenstein MW, See RE (2006). Potentiation of cue-induced reinstatement of cocaine-seeking in rats by the anxiogenic drug yohimbine. Behav. Brain Res.

[b14] Funk D, Li Z, Shaham Y, Le AD (2003). Effect of blockade of corticotropin-releasing factor receptors in the median raphe nucleus on stress-induced c-fos mRNA in the rat brain. Neuroscience.

[b15] Gianoulakis C, de Waele JP, Thavundayil J (1996). Implication of the endogenous opioid system in excessive ethanol consumption. Alcohol.

[b17] Grilli M, Neri E, Zappettini S, Massa F, Bisio A, Romussi G (2009). Salvinorin A exerts opposite presynaptic controls on neurotransmitter exocytosis from mouse brain nerve terminals. Neuropharmacology.

[b18] Holter SM, Henniger MS, Lipkowski AW, Spanagel R (2000). Kappa-opioid receptors and relapse-like drinking in long-term ethanol-experienced rats. Psychopharmacology.

[b19] Jackson KJ, McLaughlin JP, Carroll FI, Damaj MI (2012). Effects of the kappa opioid receptor antagonist, norbinaltorphimine, on stress and drug-induced reinstatement of nicotine-conditioned place preference in mice. Psychopharmacology.

[b20] Johansen JP, Cain CK, Ostroff LE, LeDoux JE (2011). Molecular mechanisms of fear learning and memory. Cell.

[b21] Jones DN, Holtzman SG (1992). Long term kappa-opioid receptor blockade following nor-binaltorphimine. Eur. J. Pharmacol.

[b22] Karoly HC, Hutchison KE (2012). Does stress contribute to the incubation of craving?. Biol. Psychiatry.

[b23] Lam MP, Gianoulakis C (2011). Effects of corticotropin-releasing hormone receptor antagonists on the ethanol-induced increase of dynorphin A1-8 release in the rat central amygdala. Alcohol.

[b24] Land BB, Bruchas MR, Lemos JC, Xu M, Melief EJ, Chavkin C (2008). The dysphoric component of stress is encoded by activation of the dynorphin kappa-opioid system. J. Neurosci.

[b25] Le A, Shaham Y (2002a). Neurobiology of relapse to alcohol in rats. Pharmacol. Ther.

[b26] Le AD, Shaham Y (2002b). Neurobiology of relapse to alcohol in rats. Pharmacol. Ther.

[b27] Le AD, Quan B, Juzystch W, Fletcher PJ, Joharchi N, Shaham Y (1998). Reinstatement of alcohol-seeking by priming injections of alcohol and exposure to stress in rats. Psychopharmacology.

[b28] Le AD, Harding S, Juzytsch W, Watchus J, Shalev U, Shaham Y (2000). The role of corticotrophin-releasing factor in stress-induced relapse to alcohol-seeking behavior in rats. Psychopharmacology.

[b29] Le AD, Harding S, Juzytsch W, Funk D, Shaham Y (2005). Role of alpha-2 adrenoceptors in stress-induced reinstatement of alcohol seeking and alcohol self-administration in rats. Psychopharmacology.

[b30] Le AD, Funk D, Harding S, Juzytsch W, Fletcher PJ (2009). The role of noradrenaline and 5-hydroxytryptamine in yohimbine-induced increases in alcohol-seeking in rats. Psychopharmacology.

[b31] Le AD, Funk D, Juzytsch W, Coen K, Navarre BM, Cifani C (2011). Effect of prazosin and guanfacine on stress-induced reinstatement of alcohol and food seeking in rats. Psychopharmacology.

[b32] Liu X, Weiss F (2002). Additive effect of stress and drug cues on reinstatement of ethanol seeking: exacerbation by history of dependence and role of concurrent activation of corticotropin-releasing factor and opioid mechanisms. J. Neurosci.

[b33] Mague SD, Pliakas AM, Todtenkopf MS, Tomasiewicz HC, Zhang Y, Stevens WC (2003). Antidepressant-like effects of kappa-opioid receptor antagonists in the forced swim test in rats. J. Pharmacol. Exp. Ther.

[b34] Mansour A, Khachaturian H, Lewis ME, Akil H, Watson SJ (1987). Autoradiographic differentiation of mu, delta, and kappa opioid receptors in the rat forebrain and midbrain. J. Neurosci.

[b35] Mansour A, Fox CA, Burke S, Meng F, Thompson RC, Akil H (1994). Mu, delta, and kappa opioid receptor mRNA expression in the rat CNS: an in situ hybridization study. J. Comp. Neurol.

[b36] Maren S (1996). Synaptic transmission and plasticity in the amygdala. An emerging physiology of fear conditioning circuits. Mol. Neurobiol.

[b37] Marinelli PW, Funk D, Juzytsch W, Harding S, Rice KC, Shaham Y (2007a). The CRF1 receptor antagonist antalarmin attenuates yohimbine-induced increases in operant alcohol self-administration and reinstatement of alcohol seeking in rats. Psychopharmacology.

[b38] Marinelli PW, Funk D, Juzytsch W, Li Z, Le AD (2007b). Effects of opioid receptor blockade on the renewal of alcohol seeking induced by context: relationship to c-fos mRNA expression. Eur. J. Neurosci.

[b39] Marinelli PW, Funk D, Harding S, Li Z, Juzytsch W, Le AD (2009). Roles of opioid receptor subtypes in mediating alcohol-seeking induced by discrete cues and context. Eur. J. Neurosci.

[b40] Marinelli PW, Funk D, Juzytsch W, Le AD (2010). Opioid receptors in the basolateral amygdala but not dorsal hippocampus mediate context-induced alcohol seeking. Behav. Brain Res.

[b41] Mason BJ, Shaham Y, Weiss F, Le AD (2009). Stress, alcohol craving, and relapse risk: mechanisms and viable treatment targets. Alcohol.

[b42] McLaughlin JP, Marton-Popovici M, Chavkin C (2003). k Opioid receptor antagonism and prodynorphin gene disruption block stress-induced behavioral responses. J. Neurosci.

[b43] McLaughlin JP, Land BB, Li S, Pintar JE, Chavkin C (2006). Prior activation of kappa opioid receptors by U50,488 mimics repeated forced swim stress to potentiate cocaine place preference conditioning. Neuropsychopharmacology.

[b44] Meister B, Villar MJ, Ceccatelli S, Hokfelt T (1990). Localization of chemical messengers in magnocellular neurons of the hypothalamic supraoptic and paraventricular nuclei: an immunohistochemical study using experimental manipulations. Neuroscience.

[b45] Nikolarakis KE, Almeida OF, Herz A (1986). Stimulation of hypothalamic beta-endorphin and dynorphin release by corticotropin-releasing factor (in vitro). Brain Res.

[b46] Pliakas AM, Carlson RR, Neve RL, Konradi C, Nestler EJ, Carlezon WA (2001). Altered responsiveness to cocaine and increased immobility in the forced swim test associated with elevated cAMP response element-binding protein expression in nucleus accumbens. J. Neurosci.

[b47] Pradhan AA, Befort K, Nozaki C, Gaveriaux-Ruff C, Kieffer BL (2011). The delta opioid receptor: an evolving target for the treatment of brain disorders. Trends Pharmacol. Sci.

[b48] Redila VA, Chavkin C (2008). Stress-induced reinstatement of cocaine seeking is mediated by the kappa opioid system. Psychopharmacology.

[b49] Reyes BA, Drolet G, Van Bockstaele EJ (2008). Dynorphin and stress-related peptides in rat locus coeruleus: contribution of amygdalar efferents. J. Comp. Neurol.

[b50] Schank JR, Goldstein AL, Rowe KE, King CE, Marusich JA, Wiley JL (2012). The kappa opioid receptor antagonist JDTic attenuates alcohol seeking and withdrawal anxiety. Addict. Biol.

[b51] Schindler AG, Li S, Chavkin C (2010). Behavioral stress may increase the rewarding valence of cocaine-associated cues through a dynorphin/kappa-opioid receptor-mediated mechanism without affecting associative learning or memory retrieval mechanisms. Neuropsychopharmacology.

[b52] Shirayama Y, Ishida H, Iwata M, Hazama GI, Kawahara R, Duman RS (2004). Stress increases dynorphin immunoreactivity in limbic brain regions and dynorphin antagonism produces antidepressant-like effects. J. Neurochem.

[b53] Sinha R, Li CS (2007). Imaging stress- and cue-induced drug and alcohol craving: association with relapse and clinical implications. Drug Alcohol Rev.

[b54] Smith JS, Schindler AG, Martinelli E, Gustin RM, Bruchas MR, Chavkin C (2012). Stress-induced activation of the dynorphin/kappa-opioid receptor system in the amygdala potentiates nicotine conditioned place preference. J. Neurosci.

[b55] Sperling RE, Gomes SM, Sypek EI, Carey AN, McLaughlin JP (2010). Endogenous kappa-opioid mediation of stress-induced potentiation of ethanol-conditioned place preference and self-administration. Psychopharmacology.

[b56] Valdez GR, Platt DM, Rowlett JK, Ruedi-Bettschen D, Spealman RD (2007). Kappa agonist-induced reinstatement of cocaine seeking in squirrel monkeys: a role for opioid and stress-related mechanisms. J. Pharmacol. Exp. Ther.

[b57] Van Bockstaele EJ, Reyes BA, Valentino RJ (2010). The locus coeruleus: a key nucleus where stress and opioids intersect to mediate vulnerability to opiate abuse. Brain Res.

[b58] Walker BM, Zorrilla EP, Koob GF (2011). Systemic kappa-opioid receptor antagonism by nor-binaltorphimine reduces dependence-induced excessive alcohol self-administration in rats. Addict. Biol.

[b59] Walker BM, Valdez GR, McLaughlin JP, Bakalkin G (2012). Targeting dynorphin/kappa opioid receptor systems to treat alcohol abuse and dependence. Alcohol.

[b60] Weathington JM, Cooke BM (2012). Corticotropin-releasing factor receptor binding in the amygdala changes across puberty in a sex-specific manner. Endocrinology.

[b61] Yajima F, Suda T, Tomori N, Sumitomo T, Nakagami Y, Ushiyama T (1986). Effects of opioid peptides on immunoreactive corticotropin-releasing factor release from the rat hypothalamus in vitro. Life Sci.

[b62] Young EJ, Williams CL (2013). Differential activation of amygdala expression by positive and negatively valenced emotional learning conditions. Front Behav. Neurosci.

[b63] Zhou Y, Leri F, Grella SL, Aldrich JV, Kreek MJ (2013). Involvement of dynorphin and kappa opioid receptor in yohimbine-induced reinstatement of heroin seeking in rats. Synapse.

[b64] Zilles K, Qu M, Schleicher A (1993). Regional distribution and heterogeneity of alpha-adrenoceptors in the rat and human central nervous system. J. Hirnforsch.

